# Characteristics of pebble shape and the amount of pebble abrasion measured with a replica reproduced on a curling rink

**DOI:** 10.1038/s41598-024-62247-0

**Published:** 2024-05-20

**Authors:** Satoshi Yanagi, Takao Kameda, Yasuhiro Harada, Kimiteru Sado

**Affiliations:** 1 Hokkaido Kushiro Meiki Senior High School, 1-38-7 Aikoku-nishi, Kushiro, Hokkaido 085-0057 Japan; 2 Snow and Ice Research Laboratory, Kitami Institute of Technology, 165 Koencho, Kitami, Hokkaido 090-8507 Japan; 3 Optical Engineering Laboratory, Kitami Institute of Technology, 165 Koencho, Kitami, Hokkaido 090-8507 Japan; 4 Kitami Institute of Technology, 165 Koencho, Kitami, Hokkaido 090-8507 Japan

**Keywords:** Engineering, Physics

## Abstract

The shape of pebbles on a curling rink was measured using a replica of the ice surface of the rink to understand the characteristics of pebbles after being in contact with stones. We focused on pebbles with flat tops for which the average shape was 3.81 mm in diameter at the lower base, 1.16 mm in diameter at the upper surface, 0.12 mm in maximum height, and 5.4° in contact angle. A scratch of about 1 µm in depth and 40 µm in width (traces of pebbles cut by a running band at the bottom of the stone) was observed on the upper surface. The pebbles were also found to have a moderate lower base diameter that preferentially contacted the nipper or stone due to its large maximum height value immediately after formation. Experiments to determine the amount of pebble abrasion associated with the passing of stones revealed that the average height of their upper surface decreased by 1 µm and the area of the upper surface increased by 0.21 mm^2^ for each stone passing as the stone cut the pebbles.

## Introduction

Many papers have been published on the mechanism of a curling stone curling, including a report^1^ published 100 years ago. These papers have proposed theories based on the difference in friction between the left and right sides of the stones^1,2^, the difference in friction between the front and rear sides of the stones^3–8^, and the turning motion at the point of contact between the stones and the ice surface^9–11^, but there is still no established theory for the curling mechanism.

The ice surface of a curling rink has ice pebbles, which support the stones that the players release. The stone glides by about 28 m, curls by about 0.5–1.5 m, and stops. From the time the stone is launched to the time it stops, the only objects in contact with the stone are the pebbles on the ice surface. Therefore, understanding the phenomena that occur between the pebbles and the stone is important in clarifying the mechanism of stone curling.

Pebbles are spherical cap-shaped ice grains produced by spraying water droplets on a rink. A few reports describe the shape of pebbles, most of which only give an overview of the shape. A recent report^12^ on the shape of pebbles indicates that pebbles range from 2 to 6 mm in diameter and from 0.4 to 1.0 mm in height, with larger diameter pebbles having lower heights. However, the pebble shape indicated in the report includes values estimated from the volume of water droplets scattered on the ice surface and is not based on actual measurements of the pebble shape. After the ice preparation process, curling rinks contain both spherical cap-shaped pebbles and spherical segment-shaped pebbles with flat tops, i.e., pebbles with visible upper surfaces. This is because the tops of protruding pebbles are cut off by special equipment called nippers after the ice surface is frozen by spraying water droplets during the ice preparation process. Curling competitions are held on this kind of ice surface.

Scratches have been found on the upper surface of the pebbles through which the stone has passed. These scratches are the marks made when the pebbles are cut by the running band at the bottom of the stone. The curl distance of stones is reported to be dependent on the surface roughness of the stone running band^13^. Therefore, the cutting of the pebbles is closely related to the curling mechanism of stones, and understanding the detailed shape of the pebbles, especially spherical segment-shaped pebbles, is considered to be indispensable in clarifying the mechanism of stone curling.

Two methods are available for determining the shape of a pebble: direct measurement and indirect measurement using a replica. The shape of an object is examined preferably by direct measurement when there are no particular constraints. However, direct measurement of the shape of pebbles requires equipment that can be used on the rink. This limits such equipment to simple measuring equipment. In contrast, indirect measurement using replicas allows the shape of pebbles to be determined with high-precision measuring equipment installed in a room. However, the shape of pebbles has not been measured using replicas. This is probably because no appropriate method has been proposed to efficiently transfer the ice surface of the rink. For example, scratches with the stone and sweep marks have been examined using replicas^7,14^, but such examination required the replica agent to be left for 1–2 h after being applied to the ice surface to cause the replica agent to cure at a low temperature of − 5 to − 3 °C.

Recently, we proposed a method of making replicas of snow crystals using a light-curing resin^15,16^. This method is advantageous in that the microstructure of the snow crystal surface can be transferred to the replica at temperatures as low as − 20 °C. The time required for the resin to cure is about 15 min, which is sufficiently short for work efficiency. The accuracy of the replica is slightly reduced due to the curing shrinkage of the resin, but the shape of irregularities of about 1 µm is known to be transferred within the accuracy of 2–3%^16^. Therefore, the shape of pebbles is expected to be determined in detail using a replica made of light-curing resin.

In this paper, we first fabricated a replica of the ice surface of the rink using a light-curing resin and then used the replica to examine the characteristics of spherical segment-shaped pebbles with flat tops. We focused on such pebbles with flat tops because these pebbles were in contact with a nipper or a stone and can be considered to have influenced the stone’s movement. We then report the results of the abrasion of pebbles caused by the passing of the stone using replicas fabricated using a light-curing resin. These results provide fundamental knowledge that will be important for future research on stone curling mechanisms.

## Method

### Replication of pebbled ice surface

**Figure 1:**
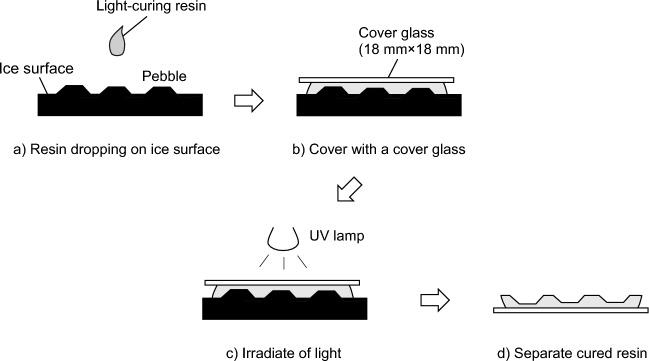
Procedure for making a replica that replicated pebbled ice surface.

Figure 1 shows the procedure for making a replica of a pebbled ice surface of a curling rink. The replica of the pebbled ice surface was made using a UV-curing resin (NOA81, Norland) as a replica agent, based on the method for making replicas of snow crystals^15^. Specifically, a) Liquid NOA81 was dropped onto the ice surface, and NOA81 was cooled to about − 7 °C by placing a bottle containing the liquid resin in a low-temperature chamber (SC-C925, Twinbird, Inc.). Next, b) the NOA81 drops were covered with a cover glass, and then the resin was cured by UV irradiation. A UV lamp (RV-1026, Repro Corporation) was used as a UV light source. The cured resin was then peeled off from the ice surface to make the replica.

The NOA81 used in the replica fabrication is a polyene polythiol UV-curing resin that is transparent before and after curing. The manufacturer indicates that the linear shrinkage of NOA81 during the curing reaction is 1.5%. For replica fabrication, 60 µl (3 drops) of NOA81 was dropped onto the ice surface. The resin was slowly cured by irradiating NOA81 with 0.5 mW/cm^2^ UV light. The duration of UV irradiation was 15 min.

The resin was cured slowly during replica fabrication to keep the temperature of NOA81 below 0 °C, which rises due to heat generated by the curing reaction, and to prevent the ice surface from melting under the curing heat. This is based on the principle of snow crystal replica fabrication using a light-curing resin^15,16^. Preliminary experiments have confirmed that NOA81 can be cured at the temperature of the resin kept below 0 °C when cured under the conditions described above.

For the NOA81 replica, as mentioned in the introduction, it is known that the shape is transferred in a reduced size mainly due to the curing shrinkage of the resin. The dimensional change rate of the replica is − 2.7% in the vertical direction and − 1.1% in the horizontal direction^15^. The dimensional change rate differs between the vertical direction and the horizontal direction because friction with the transfer surface prevents the resin from shrinking in the horizontal direction.

### Measurement of pebble shape

The replica used for measuring the pebble shape was made on August 7, 2018 at the ADVICS Tokoro Curling Hall in Kitami City, Hokkaido, Japan. Since this experiment aimed to characterize a pebble with a flat top, the replica was made by transferring the ice surface after 10 passings of a stone. The stones were released without rotation. To create pebbles at the ADVICS Tokoro Curling Hall, distilled water at 15 °C and 40 °C was sprayed on the ice rink in each direction, making one round trip in total. As equipment for spraying water droplets, #76 was used (0.51 mm in diameter) for spraying distilled water at 15 °C and #74 (0.57 mm in diameter) was used for spraying distilled water at 40 °C.

The shape of the replica was measured with a laser microscope (VK-9700, KEYENCE). First, platinum was deposited on the replica, and then 3D profile data of the replica was obtained by VK-9700. The 3D profile data was then used to measure the diameter of the lower base of the pebble in the replica *d'*_1_, the diameter of the upper surface *d'*_2_, the maximum height *h'*_m_, and the contact angle *θ'*. The measured values were then corrected based on the copying accuracy to obtain the actual lower base diameter *d*_1_, upper surface diameter *d*_2_, maximum height *h*_m_, and contact angle *θ*. If the replica showed scratches on the surface of the pebble, the shape of the scratches was also measured.

The measurement accuracy of the VK-9700 is 3*σ* = 0.020 µm in the *xy*-direction and *σ* = 0.014 µm in the *z*-direction when the supplied 50 × objective lens was used. However, the 50 × objective lens was used to measure the shape of the scratches, because the 50 × objective lens has a narrow field of view, and the 3D profile data that can be obtained in a single measurement is limited to 270 × 202 µm.

To measure the pebble shape, 3D profile data of the replica was obtained using the supplied 10 × objective lens, which provides a field of view of 1350 × 1012 µm. The measurement accuracy of the VK-9700 with the 10 × objective lens is *σ* = 0.79 µm in the *z*-direction. The measurement accuracy of the supplied 10 × objective lens was verified by ourselves using a roughness standard specimen (MT178-601, Mitutoyo) for calibration.

### Observation of pebble abrasion

To examine the amount of pebble abrasion associated with the passings of stones, the same pebbles on the ice surface were identified and replicated immediately after nipping and after 1, 5, and 10 passings of a stone, and the shapes of the pebbles were compared. The same pebbles on the ice surface were identified using close-up images of the ice surface. For examining the amount of pebble abrasion, the stone with a known surface roughness *R*_a_ of the running band was released in the experiment. In this case, the same stone A as used in Kameda et al.^13^ was used. The running band *R*_a_ of this stone was 2.772 ± 0.195 µm (Average ± SD). The stone was given a clockwise rotation and released at a velocity of about 0.5 m/s. Replicas were made on June 7, 2020 in the curling hall described in the previous section.

The amount of pebble abrasion was evaluated by determining the average height of the upper surface *h*_a_ and the area of the upper surface *s*. This is because the upper surface is created by the cutting of the pebble, and its shape is considered to reflect the amount of pebble abrasion.

For examining the amount of pebble abrasion, the shape of the replica was measured with a white interferometer (Profilm 3D, Filmetrics). The reason for this is that Profilm 3D provides a 4.0 × 3.4 mm field of view when the supplied 5× objective lens is used, allowing efficient obtaining of 3D profile data for the entire transferred pebble. When the supplied 5 × objective lens is used, the resolution of the Profilm 3D in the *xy*-direction is 2.1 µm, the resolution in the *z*-direction is 0.1 nm, and the accuracy is ± 0.7%.

To measure the shape of the replica, the 3D profile data of the replica was first obtained using the 5× objective lens supplied with Profilm 3D. Next, the data of the upper surface of the pebble was extracted from the 3D profile data, and the average height *h'*_a_ and area *s'* of the upper surface of the replica were measured from the extracted data. The measured values were then corrected based on the copying accuracy to obtain the actual height *h*_a_ and area *s*.

### Correction

The shape of the pebble determined from the 3D profile data of the replica was corrected based on the copying accuracy. When the correction was used to determine the dimension *h* perpendicular to the transfer surface, such as the maximum height *h*_m_ of the pebble or the average height *h*_a_ of the upper surface, the following Eq. (1) was used to correct the measured values:

1 $$h = \left( {1 + \frac{2.7}{{100}}} \right) \times h^{\prime}$$

where *h'* is the maximum height of the pebble *h'*_m_ or the average height of the upper surface *h'*_a_ as determined by the replica. Equation (1) corrects the replica measurements based on the fact that the dimension changes by − 2.7% in the direction perpendicular to the transfer surface. For determining the horizontal dimension *d* relative to the transfer surface, such as the lower base diameter *d*_1_ or the upper surface diameter *d*_2_ of the pebble, the following Eq. (2) was used to correct the measured values:

2 $$d = \left( {1 + \frac{1.1}{{100}}} \right) \times d^{\prime}$$

where *d'* is the diameter of the lower base *d'*_1_ or the diameter of the upper surface *d'*_2_ of the pebble as determined by the replica. Equation (2) corrects the replica measurements based on the fact that the dimension changes by − 1.1% in the horizontal direction relative to the transfer surface. Equation (2) was also used to correct the measured values when the actual area *s* was determined from the area *s'* of the upper surface in the replica.

For determining the contact angle *θ* of the pebble, we also corrected the measured value on the replica using the relational equation shown in Eq. (3) below:

3 $$\tan \theta = \left( {\frac{100 + 2.7}{{100 + 1.1}}} \right)\tan \theta^{\prime}$$

where *θ'* is the contact angle of the pebble determined using the replica. In Eq. (3), the actual contact angle *θ* is obtained using the copying accuracy in two directions: vertical and horizontal to the transcription surface.

## Results and discussion

### Shape of pebbles

**Figure 2:**
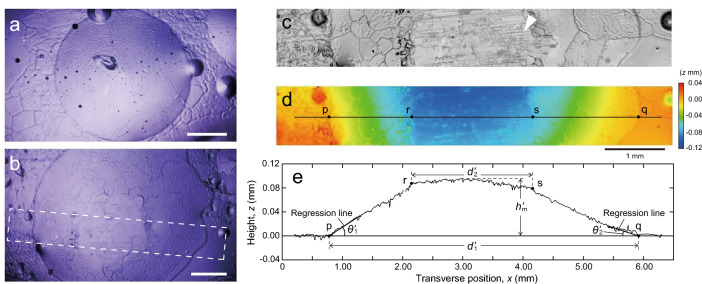
Observational results of the replica of pebble. (**a**) Optical microscopic image of the replica of pebble with spherical top, (**b**) optical microscopic image of the replica of pebble with flat top, the dotted box shows the area 3D profile data was examined, (**c**, **d**) 3D profile data of the replica shown in (**b**) with laser microscope (**c**: ultra-deep image of the replica, **d**: contour map of 3D profile data), the line shown in (**d**) indicates the line sectional profile was examined, (**e**) height *z*-inversed sectional profile curve of the replica. Points “p” and “q” indicate points used to measure the diameter of the lower base *d'*_1_. Points “r” and “s” indicate points used to measure the diameter of the upper surface *d'*_2_. In addition, regression lines used to determine the contact angles *θ'*_1_ and *θ'*_2_ are shown in (**e**). Scale bar = 1 mm.

Figure 2 shows optical microscopic images of the replica of pebbles and the measurement of the shape of the replica using a laser microscope. The replicas shown in Fig. 2 were made by the procedure shown in Fig. 1 and by transferring pebbles on the ice surface after 10 passings of a stone.

Figure 2a and b are optical microscopic images of replicas of pebbles. Figure 2a is a replica of a pebble with a spherical top, which is a spherical cap-shaped pebble formed immediately after spraying of water droplets during ice preparation. Figure 2b is a replica of a pebble with a flat top. The flat top is the upper surface created by cutting the top with a nipper or a stone. Figure 2b shows a merged image of three images taken by moving the field of view to show the entire image of the pebble. The pebble shape was measured on 14 pebbles, including the two types of pebbles with different top shapes shown in Fig. 2a and b.

The two types of pebble tops shown in Fig. 2a and b on the ice surface after 10 stone passings indicate variations in the height of the pebbles immediately after formation. The cause of the pebble formation with such different heights may be related to the fact that water droplets of different temperatures (15 °C and 40 °C) were sprayed during the ice preparation process of the rink, and the pieces of equipment with different hole diameters (#76 and #74) were used for the two types of water droplet spraying. This is because both the water temperature of the water sprayed and the diameter of the holes in the equipment used for spraying can affect the volume of the water droplets that are the source of the pebbles.

Figure 2c and d show the results of replica shape measurements using a laser microscope. Here, we show the measurement results for the replica of the pebble with a flat top shown in Fig. 2b. In Fig. 2c, the upper surface of the pebble shows transferred scratches and ice debris, indicating that the pebble was in contact with the stone. In Fig. 2d, the transferred pebble can be seen as a concave area on the replica surface. This replica can thus be used to determine the shape of the scratches as well as the shape of the pebble.

Figure 2e shows the extracted 3D profile data along the solid line in Fig. 2d. However, in Fig. 2e, the height *z* in the extracted data is inverted to represent the shape of the convex pebble on the ice surface. The missing data at around 2 mm at the transverse position is the portion excluding anomalies caused by local irregularities in the replica, which are transferred ice debris in this case. From Fig. 2e, the shape of the pebble in the replica was measured to have a lower base diameter *d'*_1_ of 5.19 mm, an upper surface diameter *d'*_2_ of 2.03 mm, and a maximum height *h'*_m_ of 0.10 mm. These measurements were corrected using Eq. (1) or Eq. (2) to obtain the actual lower base diameter *d*_1_, upper surface diameter *d*_2_, and maximum height *h*_m_.

The contact angle *θ'* of the pebble on the replica was determined by the slope of the regression line near the outer edge of the pebble. In Fig. 2e, the contact angle *θ'*_1_ near point p was found to be 3.7°, and *θ'*_2_ near point q to be 1.9°. These measurements were corrected using Eq. (3) to obtain the actual contact angles *θ*_1_ and *θ*_2_.

Table 1 shows the shapes of the 14 pebbles resulting from the correction. In this case, the shapes of 4 pebbles with spherical tops and 10 pebbles with flat tops were determined.

**Table 1:** Size of pebbles observed on ice surface after 10 passings of a stone.

No.	Diameter of lower base, *d*_1_ (mm)	Diameter of upper surface, *d*_2_ (mm)	Height, *h*_m_ (mm)	Contact angle, *θ* (°)		Top shape	Scratches
				*θ*_1_	*θ*_2_		
1	2.73	0.82	0.10	4.1	7.0	Flat	+
2	2.69	−	0.12	6.2	4.9	Spherical	−
3	2.78	0.95	0.12	7.3	7.3	Flat	+
4	5.19	2.03	0.10	3.8	1.9	Flat	+
5	3.28	0.80	0.12	4.7	4.6	Flat	−
6	3.75	1.22	0.12	5.5	6.0	Flat	+
7	3.58	−	0.14	5.1	7.0	Flat	−
8	4.30	1.24	0.12	5.1	5.2	Flat	+
9	4.42	0.40	0.12	4.7	4.3	Flat	−
10	6.34	−	0.12	2.6	3.4	Spherical	−
11	3.63	−	0.11	5.7	3.3	Spherical	−
12	2.68	0.52	0.13	6.6	7.0	Flat	+
13	2.35	−	0.12	5.7	6.4	Spherical	−
14	5.38	2.43	0.15	5.4	4.7	Flat	+
							+ : Observed − : Not observed

**Figure 3:**
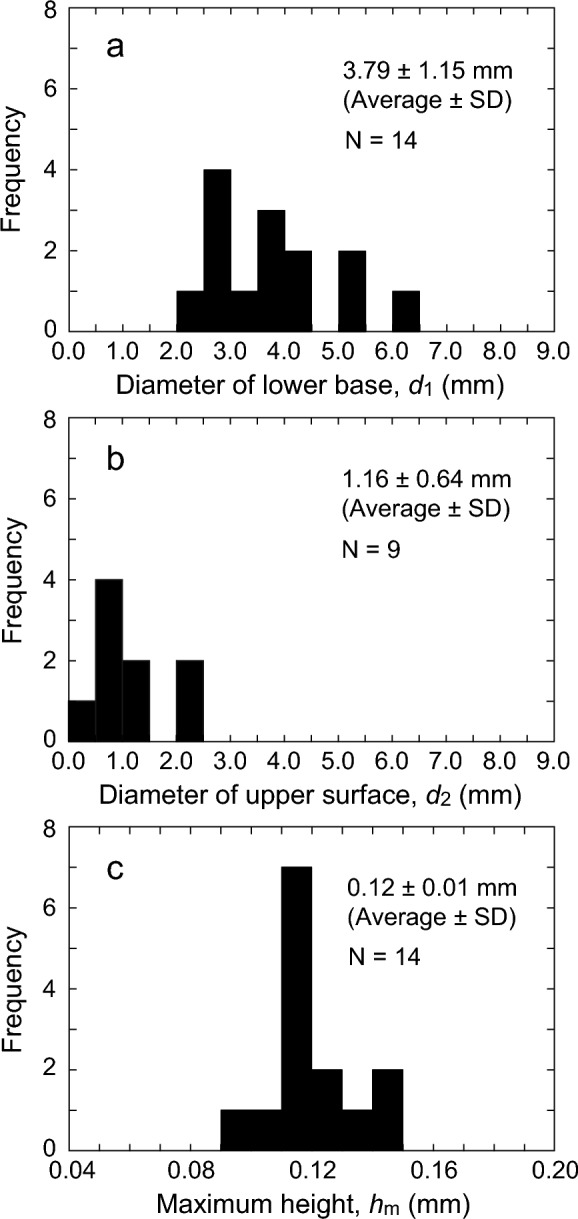
Frequency distributions of pebble size. (**a**) diameter of lower base *d*_1_, (**b**) diameter of upper surface *d*_2_, (**c**) maximum height *h*_m_.

Figure 3 shows the distribution of *d*_1_, *d*_2_, and *h*_m_ of the pebble based on the results presented in Table 1. Figure 3a shows that the lower base diameter *d*_1_ of the pebble is 3.79 ± 1.15 mm (Average ± SD). The distribution of *d*_1_ was consistent with that of the pebble diameters in the existing reports described in the introduction, i.e., 2–6 mm. The variation *σ* = 1.15 mm in the *d*_1_ of the pebbles can be attributed to the variation in the diameter of the water droplets, i.e., the volume of the droplets, spread during the ice preparation process.

Note that the pebbles with the smallest and largest *d*_1_ in Fig. 3a, i.e., No. 13 and No. 10 in Table 1, were both pebbles with spherical tops. These are pebbles without coming into contact with the nippers or stones because the maximum height *h*_m_ was relatively smaller than the surrounding pebbles. The pebbles with flat tops in contact with the nippers or stones were distributed in the range from 2.68 to 5.38 mm, excluding the minimum and maximum values. Thus, the pebble had a moderate *d*_1_ that preferentially contacted the nipper or stone due to the large maximum height *h*_m_ when the top was spherical immediately after formation.

Figure 3b shows that the upper surface diameter *d*_2_ of the pebble is 1.16 ± 0.64 mm (Average ± SD). For all pebbles, the upper surface diameter *d*_2_ was smaller than *d*_1_, and on average *d*_2_ was about 30% of *d*_1_.

The upper surface diameter *d*_2_ of the pebble is considered to be an important parameter for stone motion^8,12^. This is because this diameter corresponds to the distance that the running band of the stone moves in contact with the pebble and this value can be used to estimate the amount of energy exerted by the stone on the pebble. However, until now, the upper surface diameter *d*_2_ of the pebble has yet to be determined accurately. Therefore, the results shown in Fig. 3b should help improve the accuracy of stone motion simulation.

Figure 3c shows that the maximum height *h*_m_ of the pebble is 0.12 ± 0.01 mm (Average ± SD), with a clear peak in the maximum height *h*_m_ of 0.11–0.12 mm. Even when we focus on the 10 pebbles with flat tops, the mean value of *h*_m_ is 0.12 mm.

In Fig. 3c, a clear peak was observed, which can be attributed to the fact that the nipper or stone cut the protruding pebble, and the value of *h*_m_ was aligned at about 0.12 mm. However, the value *h*_m_ of the pebble in Fig. 3c is smaller than the previously estimated pebble height (0.4–1.0 mm)^12^. Therefore, the pebble height is smaller than previously recognized, and is about 0.12 mm on the ice surface where the stone made 10 passings.

### Contact angles

**Figure 4:**
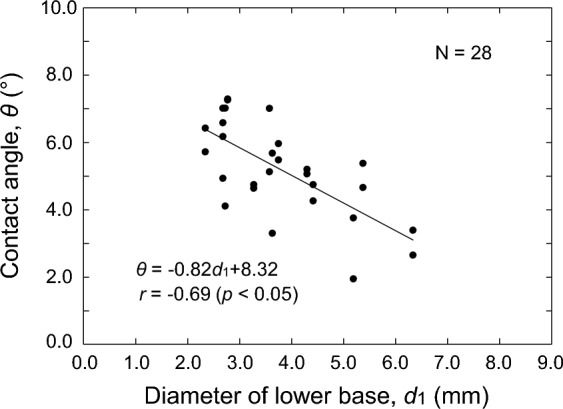
Relationship between contact angle *θ* and diameter of lower base *d*_1_.

The contact angle *θ* of the pebble is 5.2 ± 3.1° (Average ± SD) in Table 1. A negative correlation was observed between the contact angle *θ* and the lower base diameter *d*_1_. Figure 4 shows a scatter plot of the relationship between *θ* and *d*_1_. The correlation coefficient *r* of the regression line was − 0.69, which was significant at 5% using a *t*-distribution test.

The reason for the negative correlation between *θ* and *d*_1_ of the pebble as shown in Fig. 4 is considered that the lower base diameter *d*_1_ of the pebble measured this time was more than 2 mm. The contact angle of a water droplet is generally considered to be determined by the properties of the droplet and the medium. However, in an experiment to investigate the contact angle of water droplets on a polycarbonate plate, it was reported that the contact angle decreases depending on the diameter of the water droplet when the diameter of the droplet is 2 mm or larger^17^. In the report, the contact angle was also investigated under nearly gravity-free conditions, and in this case, the phenomenon of contact angle reduction depending on the diameter of the water droplets was not observed. Therefore, the negative correlation between *θ* and *d*_1_ of the pebble in Fig. 4 can be considered to be caused by the effect of gravity under 1G on water droplets with a diameter larger than 2 mm.

The contact angle of water on ice is reported to be 12°^18^. On the other hand, the average value of *θ* of the pebble we determined was 5.2°, which is about 7° smaller than the average value of the contact angle of water on ice. This can also be attributed to the above-mentioned effect of gravity. The pebble with the lower base diameter *d*_1_ of more than 2 mm in this study had a smaller contact angle than the water on the ice.

We stated in section “Shape of pebbles” that pebbles have a moderate value of *d*_1_ with a large value of *h*_m_ immediately after formation. We also mentioned that the moderate value of *d*_1_ ranges from 2.68 to 5.38 mm. On the other hand, Fig. 4 shows the relationship between *θ* and *d*_1_ using the regression line equation. Using this equation, we can calculate the value of *h*_m_ for *d*_1_ of the pebble and estimate the moderate value of *d*_1_. Assuming that the spherical cap-shaped pebble is a part of a perfect sphere, the maximum height *h*_m_ of the pebble can be calculated by the following Eq. (4):

4 $$h_{{\text{m}}} = \frac{{d_{1} }}{2}\tan \frac{\theta }{2}$$

where Eq. (4) is based on the half-angle method, which approximates the droplet shape as a part of a sphere and calculates the contact angle from the droplet radius and height. Substituting the regression line equation shown in Fig. 4 into Eq. (4), Eq. (5) can be obtained to calculate *h*_m_ from *d*_1_.

5 $$h_{{\text{m}}} = \frac{{d_{1} }}{2}\tan \frac{{\left( { - 0.82d_{1} + 8.32} \right)}}{2}$$

The value of *h*_m_ is calculated by Eq. (5) to be 0.09 mm when *d*_1_ is 5.0 mm, indicating a maximum value. However, the value of *h*_m_ for *d*_1_ calculated by Eq. (5) is 0.03 to 0.05 mm smaller than that of the pebble with a spherical top shown in Table 1. This means that the spherical cap-shaped pebble is not a part of a perfect sphere, but is a part of a distorted sphere with a slight bulge near the top. The reason for the distorted pebble shape needs to be confirmed by observation, but it seems to relate to the expansion of the volume due to the freezing of the water droplets.

However, the value of *d*_1_ at which *h*_m_ reaches its maximum value, i.e., 5.0 mm, as calculated in Eq. (5), is consistent with the moderate range of *d*_1_, i.e., 2.68–5.38 mm, as described in section “Shape of pebbles”. The fact that the pebble with the largest *h*_m_ in Table 1 had the lower base diameter *d*_1_ of 5.38 mm indicates that the pebble tends to have its maximum height *h*_m_ when the lower base diameter *d*_1_ is about 5 mm. Therefore, the moderate *d*_1_ at which preferential contact with the nipper or stone occurs due to the large *h*_m_ immediately after formation can be estimated to be 5.0 mm based on Eq. (5).

### Scratches

**Figure 5:**
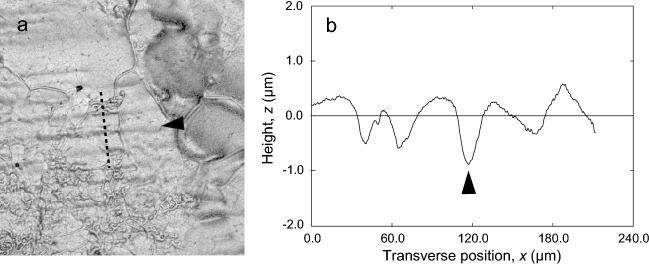
Scratches of the upper surface of a pebble. (**a**) Ultra-deep image of scratches, (**b**) height *z*-inverted sectional profile curve of the replica. Arrowheads indicate the same scratch shown in Fig. 2c.

Figure 5 shows the measurement results of the shape of scratches. Figure 5a is a magnified image of the upper surface of the replica shown in Fig. 2c. Figure 5b shows the data extracted from the dotted line in Fig. 5a. In Fig. 5b, the height *z* of the extracted data is inverted to show the shape of the scratches, which are grooves on the upper surface. In Fig. 5b, the scratches transferred to the replica were found as grooves of about 1 µm in depth and 40 µm in width. Even when these values are corrected based on the copying accuracy, the depth and width are still about 1 µm and 40 µm, respectively.

The standard size of scratches on pebbles has been reported to be a groove of 3 µm in depth and 50 µm in width^7^. In contrast, in the present measurements, the width of the scratch was similar to that previously reported, but the depth was slightly shallower, with a groove of about 1 µm.

In this study, we have shown the measurement results of a single scratch in Fig. 5, but have not examined standard widths and depths based on a large number of measurements. However, the scratches are traces of the stone cutting the pebble, and as mentioned in the introduction, the distance of the stone curling depends on the surface roughness of the running band^13^. For this reason, a detailed study of the shape of the scratches will be a subject for future research.

### Characteristics of pebble shape

**Figure 6:**
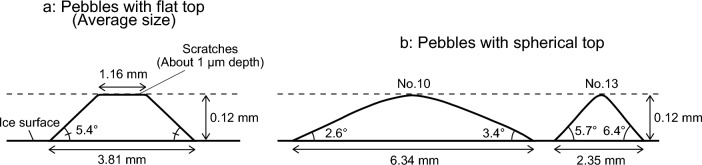
Schematic diagram of pebble shape observed on ice surface after 10 passings of a stone.

Figure 6 shows a schematic diagram of the characteristics of the pebble shape. Of the 14 pebbles whose shape was measured, the 10 pebbles with flat tops had an average diameter of 3.81 mm for the lower base *d*_1_, 1.16 mm for the upper surface *d*_2_, the average value of 0.12 mm for the maximum height *h*_m_, with the average value of the contact angle *θ* of 5.4°, as shown in Fig. 6a. These pebbles were considered to be in preferential contact with the nippers or stones because the value of *d*_1_, in other words, the volume of the original pebble droplets, was moderate and the maximum height *h*_m_ was larger than the surrounding area immediately after formation. The scratch of about 1 µm in depth and 40 µm in width was also observed on the upper surface.

Figure 6b shows the shapes of pebbles Nos. 10 and 13 in Table 1 and compares the shapes of pebbles with spherical tops and those with flat tops. The pebble with a spherical top is considered to have formed with the maximum height *h*_m_ smaller than its surroundings because the volume of the droplet from which the pebble originated was not moderate.

### Pebble abrasion

For examining the amount of pebble abrasion, as described in section “Observation of pebble abrasion”, the same pebble on the ice surface immediately after nipping and after 1, 5, and 10 passings of a stone was transferred to the replica. When the replicas were observed under an optical microscope, the upper surface was recognized on the pebble after the first stone passing. Therefore, the amount of pebble abrasion was examined using replicas of the pebble after the first stone passing.

**Figure 7:**
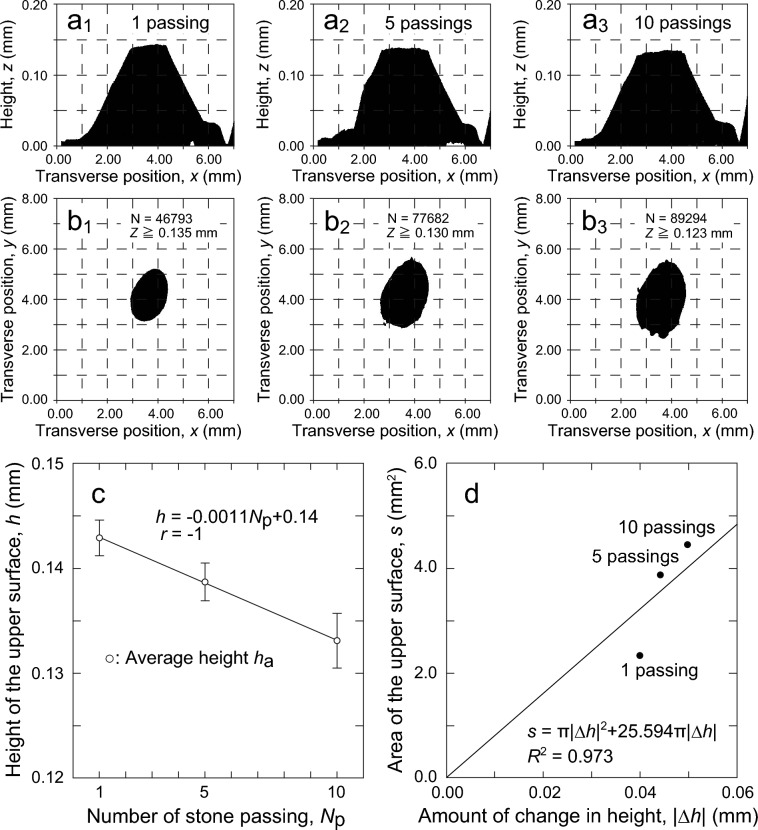
Change in the pebble shape with the stone passings observed in the replica. (**a**_**1**_**–a**_**3**_) Projection diagrams of height *z*-inversed 3D profile data of the replica of the same pebble onto the *xz* plane, (**b**_**1**_**–b**_**3**_) projection diagrams onto the *xy* plane showing the area of the upper surface, (**c**) change in the average height of the upper surface *h*_a_ for the number of a stone passing, the error bar indicates SD. (**d**) Area of the upper surface *s* for the amount of change in height |Δ*h*| compared with the pebble before stone passing that had been spherical top.

Figure 7 shows the 3D profile data of the replica of the pebble measured by the white interferometer, and the average height of the upper surface *h*_a_ and the area of the upper surface *s* obtained by the method described in section “Observation of pebble abrasion” using the 3D profile data. Figure 7a1–a3 show the 3D profile data of the replica of the same pebble on the ice surface after 1, 5, and 10 passings of a stone, respectively. Here, the *z*-inverted 3D profile data is projected onto the *xz* plane to show the shape of the pebble, which is the convex part of the ice surface. From the 3D profile data, the data of the upper surface was extracted.

Figure 7b1–b3 show the extracted upper surface data. When extracting the data for the upper surface, we extracted the data for the area near the top of the pebble, where the change in height is smaller. For this purpose, we used the height distribution in the 3D profile data. In Fig. 7b1, Fig. 7b2, and Fig. 7b3, data with *z* greater than 0.135 mm, 0.130 mm, and 0.123 mm, respectively, were extracted and projected to the *xy* plane.

From the extracted data, the average height *h'*_a_ and area *s'* of the upper surface of the replica were calculated. The average height *h'*_a_ of the upper surface was calculated as the average value of *z* values in the extracted data. The area *s'* of the upper surface was calculated as *s'* (mm^2^) = N × 6.979^2^ × 10^–6^ based on the number of measurement points N and the sampling interval in the *xy*-direction (6.979 µm) of the extracted data. The calculated values were corrected using Eq. (1) or Eq. (2) to obtain the actual average height *h*_a_ and area *s*.

The replica of the pebble immediately after nipping was used to determine the maximum height *h*_m_ of the pebble with a spherical top before the upper surface was formed. The maximum height *h*_m_ of the pebble with a spherical top was determined by inverting the height *z* of the 3D profile data of the replica measured with a white interferometer and then correcting the maximum value of *z* in the inverted data using Eq. (1).

Figure 7c shows the average height *h*_a_ of the upper surface. The average height *h*_a_ of the upper surface was 0.143 ± 0.002 mm (Average ± SD) for the pebble after 1 passing of a stone, but decreased to 0.139 ± 0.002 mm (Average ± SD) after 5 passings of a stone and to 0.133 ± 0.003 mm (Average ± SD) after 10 passings of a stone.

Figure 7d shows the area of the upper surface *s*. In Fig. 7d, the absolute value of the change in the height of the pebble with the passing of a stone |Δ*h*| is used as the *x*-axis, and *s* is shown for |Δ*h*|. |Δ*h*| was calculated as |Δ*h*| =|*h*_a_ − *h*_m_| based on the fact that the maximum height *h*_m_ of the pebble immediately after nipping was 0.183 mm. This is because we considered that if the shape of a pebble with a spherical top is a spherical cap that is a part of a sphere, the area of the upper surface *s* can be approximated by the bottom area of a spherical cap with the top height of |Δ*h*|. The values of |Δ*h*| for the pebble after 1, 5, and 10 passings of a stone were 0.040 mm, 0.044 mm, and 0.050 mm, respectively.

In Fig. 7d, the area of the upper surface *s* was 2.33 mm^2^ for the pebble after 1 passing of a stone, increasing to 3.87 mm^2^ after 5 passings of a stone and 4.45 mm^2^ after 10 passings of a stone. These values were regressed by the least-squares method on the equation (*s* = π|Δ*h*|^2^ + 2π*R*_s_|Δ*h*|), which represents the bottom area of the spherical cap with the top height of |Δ*h*| that is a part of a sphere with a radius of *R*_s_. The regression equation shown in Fig. 7d was obtained. The coefficient of determination *R*^2^ of the regression equation is 0.973, which is a good fit. Therefore, the values of |Δ*h*| and *s* shown in Fig. 7d, i.e., the values of *h*_a_ and *s* obtained from the 3D profile data of the replica, indicate the shape change caused by the cutting of the spherical cap-shaped pebble from the top side.

Therefore, we compared the values *h*_a_ and *s* of the pebble after 1 passing of a stone and after 10 passings of a stone to determine the amount of change per stone passing. The results showed that *h*_a_ decreased by 1 µm and *s* increased by 0.21 mm^2^ for each stone passing. In other words, the amount of abrasion of the pebble due to the passing of the stone can be determined.

## Conclusions

1. The average shape of a spherical segment-shaped pebble that has a flat top on a curling rink is 3.81 mm in diameter at the lower base, 1.16 mm in diameter at the upper surface, 0.12 mm in maximum height, and 5.4° in contact angle. This is the shape of the pebble in contact with the nipper or stone.
2. The pebble has a moderate lower base diameter with a large maximum height immediately after formation.
3. The upper surface of the spherical segment-shaped pebble has scratches of about 1 µm in depth and 40 µm in width.
4. The average height of the upper surface of the pebble decreases by 1 µm and the area of the upper surface increases by 0.21 mm^2^ with each stone passing.

## Data Availability

The datasets generated from this study are available from the corresponding author on reasonable request.
